# Aqueous humor hepcidin prohormone levels in patients with primary open angle glaucoma

**Published:** 2010-09-07

**Authors:** Rana Sorkhabi, Amir Ghorbanihaghjo, Alireza Javadzadeh, Behzad Fallahi Motlagh, Solmaz Shoa Ahari

**Affiliations:** 1 Department of Ophthalmology, Tabriz University of Medical Sciences, Tabriz, Iran; 2 Biotechnology Research center, Tabriz University of Medical Sciences, Tabriz, Iran

## Abstract

**Purpose:**

This study was designed to evaluate the levels of Interleukin-6 (IL6) and Hepcidin prohormone (Hep) in the serum and aqueous humor of patients with primary open-angle glaucoma (POAG), as well as those with senile cataract as a control group.

**Methods:**

Levels of IL6 and Hep were measured by enzyme-linked immunosorbent assay (ELISA) methods in serum and aqueous humor aspirates taken from 45 patients (POAG=20, Control=25) during anterior segment surgery.

**Results:**

The mean aqueous humor Hep concentration in eyes with POAG was significantly higher than that controls (34.55±23.01 ng/ml versus 20.82± 24.63 ng/ml, p=0.04). There was no significant difference between the serum Hep concentration of POAG and the control group (359.46±113.03 ng/ml versus 287.36±130.53 ng/ml, p=0.08). There was also no significant difference between either serum (6.18±5.22 versus 10.84±3.37, pg/ml p=0.112) or aqueous humor (4.39±3.06 versus 5.79±2.63, pg/ml p=0.14) IL6 concentrations of POAG and the control groups. No significant correlations were found between aqueous humor and serum Hep and IL6 levels in either POAG or the control groups (p>0.05).

**Conclusions:**

The aqueous humor Hep level may exist locally and independent from the IL6 increase in patients with POAG, suggesting that Hep might represent a bridge protein between local inflammation and the consequent loss of retinal ganglion cells.

## Introduction

Glaucoma is a multifactorial, progressive optic neuropathy with a characteristic loss of retinal ganglion cells (RGCs) beyond typical age-related baseline loss. It is the second leading cause of blindness in the word [1-3].

Reactive oxygen species (ROS) play a fundamental role in the pathophysiology of many diseases, including glaucoma. The iron-catalyzed formation of ROS is a major player in these processes. The oxygen radical superoxide is a product in cells from oxidized reactions in the mitochondria and other redox reactions in cells. Superoxide is detoxified by superoxide dismutase with the resulting formation of hydrogen peroxide, which in the presence of ferrous iron can form the highly reactive and damaging hydroxyl radical. Clearly, careful control of iron availability is central to the maintenance of normal cell functioning [4].

The Hepcidin prohormone (Hep) is a small peptide produced in the liver. Human Hep is produced from an 84 amino acid precursor, including a putative single peptide. Hep is an important peptide hormone that plays a critical role in the regulation of iron efflux from numerous cell types, including intestinal cells, macrophages, and hepatocytes. Hep binds to and induces the degradation of ferroportin, a protein for iron efflux [5]. Therefore, Hep is responsible for iron homeostasis, decreasing iron uptake from the intestine, and release from the liver in conditions of iron overload. Conversely, Hep syntheses are decreased in iron deficiency, resulting in increased iron uptake from the intestine and release from liver stores [6].

A recent study found that Hep is expressed in Muller cells, photoreceptor cells, and retinal pigmented epithelium (RPE) in an expression pattern similar to that of ferroportin. The increase in Hep expression correlates with a decrease in ferroportin expression, as well as an increase in oxidative stress and apoptosis, as would be expected from an increase in intracellular iron resulting from decreased iron export [7].

The expression of Hep in the retina points to the local intraocular regulation of iron metabolism, separate from a dependence on the circulation liver-derived hormone: circulating Hep would likely be inaccessible to intraocular tissues due to the presence of blood-ocular barriers [8].

Interleukin-6 (IL6) is a multi-functional cytokine that regulates immune responses, acute phase reactions, and hematopoiesis, and may play a central role in host defense mechanisms. Studies have identified IL6 as a possible inducer of Hep syntheses [9-11].

Based on the reported literature, the aim of the present study was to investigate the Hep and IL6 levels in the aqueous humor and serum of patients with primary open-angle glaucoma (POAG) and compare these with senile cataract patients as a comparative (control) group.

## Methods

The study was designed as a prospective case control study and was performed from August 2008 to July 2009 in the Nikookari Eye Hospital in Tabriz, Iran.

Twenty patients with POAG (9 females and 11 males) and intraocular surgery indication, and 25 age- and sex-matched senile cataract patients without glaucoma (13 males and 12 females) were included in the study.

Informed consent was taken from all of the patients, and the study was approved by the ethical committee of Tabriz University of Medical Science and conducted in accordance with the ethical principles outlined in the Declaration of Helsinki.

Participants in the POAG group were recruited from patients who required trabeculectomy or phacotrabeculectomy for uncontrolled or the progression of glaucomatous optic damage on maximally tolerated medication. Six patients from this group underwent phacotrabeculectomy and 14 patients underwent trabeculectomy.

Participants were excluded from the study for a history of laser surgery or intraocular surgery, major systemic diseases (diabetes, renal or hepatic disease, hematologic and autoimmune disorders, arteriosclerotic disease), and any history of special drug usage (e.g., iron preparations, chemotherapeutic agents, vitamins) that could influence the Hep and IL6 levels.

The intraocular pressure (IOP), measured by Goldmann applanation tonometry, was 21 mmHg or higher in the POAG group. All patients had typical glaucomatous optic disc damage on fundoscopy and a visual field defect was noted in all patients with the full threshold or Swedish Interactive Threshold Algorithm strategy, program 24–2, visual field test on the Humphrey Field Analyzer.

The cup-to-disc ratio of all patients was ≥0.6(0.72±0.20). Angles were wide open on gonioscopy. According to the cup-to-disc ratio, glaucoma patients were subdivided into the moderate and severe stage subgroups. In the moderate stage subgroup, the cup-to-disc ratio was ≤0.7. All patients received at least two topical antiglaucoma drugs.

All patients enrolled in the control group had senile cataracts with a normal IOP and did not receive any topical medication.

Aqueous humor samples (≈0.2 ml) were collected at the beginning of surgery through clear corneal paracentesis, using a 27 gauge needle on an insulin syringe under an operating microscope with special care to avoid blood contamination. Aqueous humor samples were immediately cooled at −70 °C and protected from light until analysis. Venous blood samples were also collected in the fasting state, 24 h before surgery. The samples were centrifuged within 1hr and stored at −70 °C until analysis was performed.

The Hep and IL6 levels in both serum and aqueous humor samples were measured by enzyme-linked immunosorbent assay (ELISA) using the DEMEDITEC GmbH (Lot: RN- 24429; Kiel-Wellsee, Germany) and Bender MedSystems GmbH (Lot: 233737; Vienna, Austria) kits, respectively.

All of the data were entered to the SPSS software package version 13 for Windows (SPSS Ins, Chicago, IL) for statistical analysis. Results are expressed as mean±SD, numbers, and their percent when appropriate. The Mann–Whitney U-test was used for statistical analyses. Correlation was evaluated by Spearman's test and p<0.05.was considered significant.

## Results

All of the patients completed the study and none were excluded. All measurements were performed successfully, without any failure in determining Hep and IL6 owing to insufficient sampling or any other complication during the analyses. Demographic and baseline characteristics of the patients are shown in Table 1. There were no statistically significant differences between the two groups in terms of age, sex, or body mass index (p>0.05 in all cases).

**Table 1:** Demographic and baseline characteristics of both study groups.

**Variable**	**POAG^(a)^ (n=20)**	**Control^(b)^ (n=25)**	**p-value**
Age (years)^(c)^	71.1±6.3	70.8±6.5	0.97^(d)^
Sex n (%)			
Female	9 (45%)	12 (48%)	0.56^(e)^
Male	11 (55%)	13 (52%)	
BMI (kg/m2^)(f)(c)^	25.56±4.26	25.35±4.12	0.52^(d)^
Intraocular pressure (mm Hg)	25.50±3.73	14.23±1.24	0.04^(d)^
Medical therapy	n (%)		
2 drops^(g)^	6 (30%)	-	
3 drops^(h)^	6 (30%)	-	
4 drops^(i)^	8 (40%)	-	
Cup-to-disc ratio (% of patients)	0.6 (30%)	-	
	0.7 (15%)	-	
	0.8 (20%)	-	
	≥0.9 (35%)	-	

^(a)^Primary open-angle glaucoma. ^(b)^Senile cataract patients. ^(c)^Data are expressed as mean±SD ^(d)^Performed by Mann–Whitney U test. ^(e)^Performed by χ^2^. ^(f)^Body mass index. ^(g)^Timolol + Latanoprost. ^(h)^Timolol + Dorzolamide + Latanoprost. ^(i)^Timolol + Dorzolamide + Latanoprost + Brimonidine.

Although the mean aqueous humor Hep concentration in patients with POAG was significantly higher than in controls (p=0.04), there was no significant difference in serum Hep concentration when compared with controls (p=0.08).

No significant difference was found in aqueous and serum IL6 concentrations in patients with POAG compared with controls (p=0.14 and p=0.11, respectively; Table 2).

**Table 2:** Comparison of the Hep and IL6 concentrations in the serum and aqueous humor of the study groups.

**Variables**	**Hep (ng/ml)**			**IL6 (pg/ml)**		
Study groups	POAG^(a)^ (Mean±SD)	Control^(b)^ (Mean±SD)	p value^(c)^	POAG^(a)^ (Mean±SD)	Control^(b)^ (Mean±SD)	p-value^(c)^
Serum	359.46±113.03	287.36±130.53	0.08	6.18±5.22	4.39±3.06	0.11
Aqueous humor	34.55±23.01	20.82±24.63	0.04	10.84±3.37	5.79±2.63	0.14

^(a)^Primary open-angle glaucoma. ^(b)^Senile cataract patients. ^(c)^Performed by Mann–Whitney U-test. IL6: Interleukin-6; Hep: Hepcidin prohormone.

There were no significant correlations between Hep and IL6 levels among patients with POAG and the control group (p>0.05) (Table 3).

**Table 3:** Correlations of aqueous humor and serum Hep and IL6 concentrations among study groups.

**Correlation**٭	**p**	**r**
**Primary open-angle glaucoma (n=20)**		
Aqueous humor Hep with serum Hep	0. 93	0.022
Aqueous humor Hep with aqueous humor IL6	0.42	−0.194
Aqueous humor IL6 with serum IL6	0.50	0.160
Serum Hep with serum IL6	0.06	−0.432
**Controls (n=25)**		
Aqueous humor Hep with serum Hep	0.06	0.378
Aqueous humor Hep with aqueous humor IL6	0.20	0.263
Aqueous humor IL6 with serum IL6	0.22	−0.254
Serum Hep with serum IL6	0.52	−0.134

*Performed by Spearman's test. IL6: Interleukin-6; Hep: Hepcidin prohormone.

Of 20 patients with POAG, only 2 patients had IOP >23 mmHg after surgery with two antiglaucoma drugs; these cases were considered failed surgical outcomes.

There were no statistically significant differences between the moderate and severe stage glaucoma subgroups in the aqueous levels of Hep and IL6 (p=0.65 and p=0.94, respectively), but the serum levels of Hep were higher in severe stage patients (p=0.01; Table 4).

**Table 4:** Characteristics of two glaucoma subgroups.

**Variable**	**Moderate POAG^(a)^ (n=9)**	**Severe POAG^(a)^ (n=11)**	p-value*
IOP (mm Hg)^(b)^	26±3.32	25.09±4.16	0.62
MD^(c)^	−15.87±8.35	−19.56±5.64	0.27
PSD^(d)^	6.29±3.64	7.94±2.54	0.34
Serum Hep (ng/ml)	291.42±77.22	422.04±107.26	0.01
Serum IL6(pg/ml)	7.82±7.56	4.56±1.73	0.67
Aqueous Hep (ng/ml)	28.19±24.60	31.40±20.06	0.65
Aqueous IL6 (pg/ml)	6.80±2.42	14.60±17.40	0.94

*Mann–Whitney U test; ^(a)^POAG: Primary open-angle glaucoma; ^(b)^IOP: Intraocular pressure; ^(c)^MD: Mean deviation; ^(d)^PSD: Pattern standard deviation; IL6: Interleukin-6; Hep: Hepcidin prohormone.

## Discussion

Conventional glaucoma therapy has been focusing on lowering IOP. Recent studies have suggested that glaucoma is caused by multiple factors that ultimately lead to the apoptotic death of retinal ganglion cells and subsequent optic nerve atrophy. Among these are changes in the microcirculation of the optic nerve head, deprivation of trophic factors, and the release of neurotoxin agents within the retina, such as glutamate, nitric oxide, and free radicals [12].

New discoveries of iron metabolism in the eye include the regulation of glutamate production and secretion [13,14], glutathione (GSH) synthesis [15], and the activity of hypoxia inducible factor-1 (HIF-1). HIF-1 has been shown to have either a clinically or experimentally mediating or contributing role in several oxygen-dependent retinal diseases such as glaucoma [16-18]. In the literature, there is a wealth of evidence linking the oxidation of proteins to cataract formation, and iron has a central role in catalyzing free radical reactions, leading to oxidative damage [19]. Iron catalyzed reactions have been linked to changes in lens DNA damage and cataract formation [20,21]. Thus, we included senile cataract patients as a comparative or control group and matched two groups of patients regarding the severity of their cataracts.

Retinal iron levels increase with age in both humans [22] and rodents [23], and with disease [24]; thus, in this study we matched the two groups regarding age and excluded all patients with a history of major systemic disease.

Alterations in the levels of proteins involved in iron metabolism in the neural retina of aging rodents suggest that deregulation of the iron metabolism and the resulting accumulation of iron could be a causative factor in age-related retinal degeneration [23].

To the best of our knowledge, this is the first study evaluating Hep levels in human aqueous humor. Gnana Parksam et al. [9] found that Hep is expressed in Müller cells, photoreceptors, and RPE in an expression pattern similar to that of ferroportins.

In our study, the concentration of Hep in the aqueous humor of the POAG group was significantly higher than that of the control group, but there was no significant increase or correlation in the serum levels. We do not know whether this phenomenon has a primary role in glaucomatous optic nerve damage or is secondary to a cascade of events that begins with high IOP and then continues to cause iron metabolism deregulation and additive damage in this way.

We also found that serum Hep levels are higher in more advanced glaucoma patients but, due to the small sample size of POAG patients, we classified these patients only according to cup-to-disc ratio. Thus, we must interpret our findings with caution.

The results of the present study suggest that local Hep secretions may have a pathogenic role in POAG. Furthermore, we did not find any correlation between IL6 and Hep levels either in serum or in aqueous humor, supporting the hypothesis that the mechanism of aqueous humor Hep regulation may be independent in serum and aqueous humor IL6 levels. A larger sample size, as well as new markers for iron metabolism, will be of great interest in terms of confirming our results. We also need to mention some limitations of our study, which will guide future studies. It must be kept in mind that some protein levels may increase in glaucoma due to disturbance in the aqueous humor outflow. Another limitation is the effect of antiglaucomatous drugs on free radical–induced apoptosis, as well as the possible effect of these drugs on Hep and IL6 levels.

We believe that understanding iron metabolism in the eye is essential in light of the preponderance of evidence to the effect that deregulation of iron metabolism occurs in neurologic and retinal degeneration, such as in glaucoma. Unfortunately, very little is known about iron metabolism in ocular tissues, and the study was a pilot study on a small group; still, our results indicate that Hep may also play a role in glaucomatous pathology.

In summary, these results indicate that aqueous humor Hep is higher in POAG patients, and future studies are required to confirm the direct etiological role of Hep in the induction of POAG.
